# The Link between Protein Kinase CK2 and Atypical Kinase Rio1

**DOI:** 10.3390/ph10010021

**Published:** 2017-02-07

**Authors:** Konrad Kubiński, Maciej Masłyk

**Affiliations:** The John Paul II Catholic University of Lublin, ul. Konstantynów 1i, 20-708 Lublin, Poland

**Keywords:** protein kinase CK2, protein kinase Rio1, phosphorylation, protein-protein interaction, benzimidazoles

## Abstract

The atypical kinase Rio1 is widespread in many organisms, ranging from Archaebacteria to humans, and is an essential factor in ribosome biogenesis. Little is known about the protein substrates of the enzyme and small-molecule inhibitors of the kinase. Protein kinase CK2 was the first interaction partner of Rio1, identified in yeast cells. The enzyme from various sources undergoes CK2-mediated phosphorylation at several sites and this modification regulates the activity of Rio1. The aim of this review is to present studies of the relationship between the two different kinases, with respect to CK2-mediated phosphorylation of Rio1, regulation of Rio1 activity, and similar susceptibility of the kinases to benzimidazole inhibitors.

## 1. Introduction

Protein kinases play important roles in key cellular processes, including the cell cycle, metabolism, and cell death [1,2]. The protein kinase superfamily with its 518 members comprises one of the largest protein superfamilies identified in the human genome [3]. In addition to their key roles in cell physiology, several protein kinases are linked to pathological states, including cancer [4,5]. This fact makes kinases attractive targets for therapeutic interventions.

**Figure 1:**
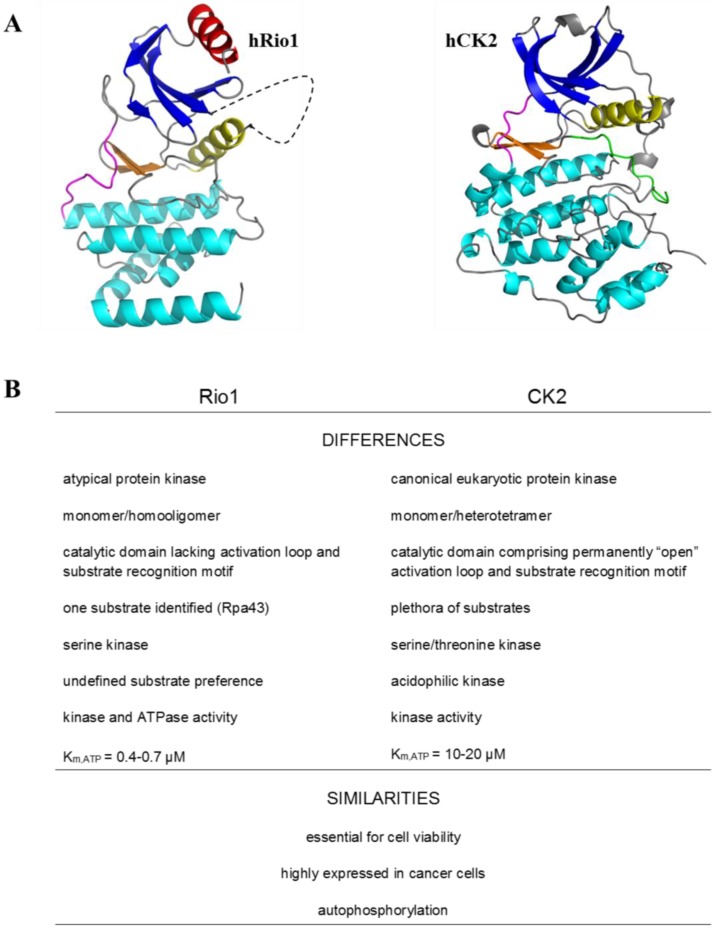
Differences and similarities between protein kinases Rio1 and CK2 [6,7,8,9,10,11,12,13,14,15,16]. (**A**) Comparison of the 3D structures of hRio1 and hCK2α visualised in PyMOL. The N-terminal β-sheets are coloured blue, the β-hairpin is coloured orange, the αC-helix is coloured yellow, the hinge is coloured magenta, the C-lobe α-helices are coloured cyan, the αR-helix is coloured red, the “flexible loop” is represented by blacked dashed line, and the activation loop is coloured green. (**B**) Comparison of other features of the two kinases.

The objective of the review article is to present a representative of the RIO family, namely the atypical protein kinase Rio1. The special intention is to emphasize the relationship of Rio1 from humans and yeast, with probably the most pleiotropic cellular kinase—CK2. Although the interaction between these two remarkably different kinases (Figure 1) has not been widely reported to date, we focus on some common aspects of their activity, namely protein-protein interaction, CK2-mediated Rio1 phosphorylation, and similar susceptibility to some benzimidazoles.

## 2. Protein Kinase Rio1

RIO kinases comprise an atypical kinase family composed of four subfamilies: Rio1, Rio2, RioK3, and RioB. The members of the RIO family are evolutionarily conserved and are present in all three domains of life. RIO kinases exhibit a trimmed version of the domain present in the canonical eukaryotic protein kinases, which lack the activation loop and the substrate recognition region [7,17,18]. To date, only one crystal structure of human Rio1 has been developed (PDBID:4OTP). Like canonical eukaryotic kinases (ePKs) (including CK2), Rio1 consists of two main lobes called the N-terminal and C-terminal lobes. The N-terminal lobe consists of a 5-stranded antiparallel β-sheet (β1-5) and a single α-helix (αC), which is located between β3 and β4.

The N-terminal lobe is connected via a “hinge region” to a C-terminal lobe, containing three α-helices (αE, αF, and αG) and a β-hairpin (β6-7) [12]. In contrast to canonical ePKs, the N-terminal lobe of Rio1 contains a RIO-kinase-specific α-helix called αR. Another specific structural feature of Rio1 is the presence of a “flexible loop” located between β3, and the density of this region is not observed in the crystal structure of hRio1. Moreover the Rio1 structure lacks the activation or the “APE” loop and the substrate recognition motif located within subdomain VIII of the canonical ePKs C-terminal lobe [7,12,19] (Figure 1).

The enzyme is involved in processes critical for cellular proliferation, including ribosome biogenesis, cell cycle progression, and chromosome maintenance [12,20,21]. Both in yeast and in humans, Rio1 is required for the last cytoplasmic step of processing 20S pre-rRNA to mature 18S rRNA, i.e. the RNA component of the 40S ribosome subunit [12,22,23,24]. Depletion of Rio1 results in strong cytoplasmic accumulation of 20S pre-rRNA, a severe decrease in the 18S rRNA level, and finally cell cycle arrest [20]. Moreover, in humans, the kinase activity of the enzyme is essential for the recycling of endonuclease hNob1 and its binding partner hDim2 from cytoplasmic pre-40S and the translocation of the proteins into the nucleus [24]. As far as the process of ribosome biogenesis is concerned, Rio1 can act as a kinase as well as ATPase. Studies on yeast have revealed that Rio1`s ATPase activity is required for late 40S biogenesis to regulate its dynamic association with pre-40S particles [12].

While the cytoplasmic functions of Rio1 are well characterized, little is known about its role in the nucleus, where it is also present. Recent studies have revealed that, Rio1 downregulates RNA polymerase I (Pol I) during anaphase by targeting its subunits Rpa43, thereby causing the polymerase to dissociate from rDNA; thus, Rio1 integrates rDNA replication and segregation with ribosome biogenesis [13].

Interestingly, the protein kinase Rio1 is characterized as an essential enzyme involved in cell viability; on the one hand; on the other hand only one physiological protein substrate (Rpa43) for this kinase is known. There are several proteins that undergo Rio1-mediated phosphorylation *in vitro*, namely, myelin basic protein, casein, histones H1 and H2A, enolase, Tomato mosaic virus MP, and the recently discovered subunit of RNA Pol I—Rpa43 [6,8,10,13,25].. The enzyme exhibits a capability of autophosphorylation at serine residues, but the physiological significance of the modification remains elusive [6,8]. It has been reported that autophosphorylation reduces oligomerization of Rio1 and promotes the most active monomeric form of the kinase [10].

## 3. Rio1 as an Interacting Partner and Target for CK2

**Figure 2:**
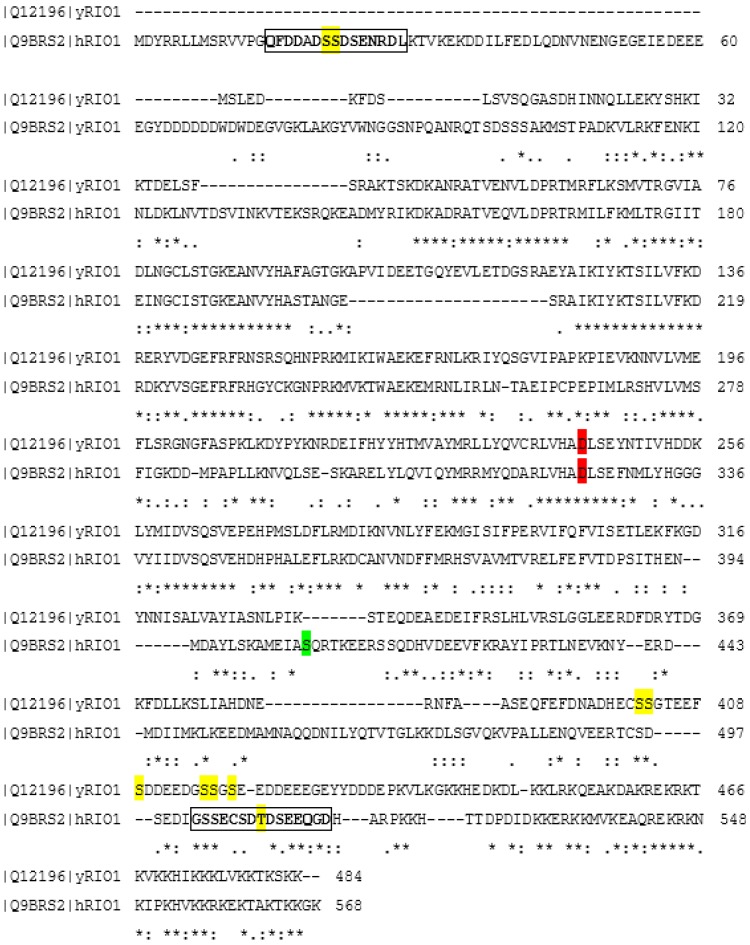
Alignment of human (Q9BRS2) and yeast Rio1 (Q12196). The alignment was performed using a Clustal Omega tool on the uniprot.org website. CK2 phosphorylation sites are marked in yellow, catalytic amino acid residues are marked in red, and the autophosphorylation site is marked in green. Two putative CK2-phosphorylation sequences in hRio1 are marked with boxes. Identical amino acids are marked with asterisks, similar amino acids are marked with colons, and different amino acids are marked with dots.

There are 18 phosphorylated amino acids within the hRio1 sequence listed at PhosphoSitePlus (www.phosphosite.org). All the sites mentioned have been revealed by High Throughput Screening, but not confirmed by site-specific methods. Among all these phosphosites, there are three (S21, S22, and T509) located within two sequences that fulfil the minimal consensus for phosphorylation by protein kinase CK2 [11] (Figure 2). Another phosphoproteome analysis project (PHOSIDA) revealed that hRio1 undergoes phosphorylation within the sequence QFDDAD**S21S22**DSENRDL [26,27]. In turn our recent unpublished data have confirmed serines 21 and 22 as the only sites modified in vitro within the human kinase Rio1 by protein kinase CK2 (Figure 2).

Interestingly, protein kinase CK2 was the first interaction partner of Rio1, identified in yeast. By means of co-immunoprecipitation, Angermayr and coworkers revealed that Rio1 preferentially interacted with CK2α´, but not with the remaining yeast CK2 subunits: CK2α, CK2β, and CK2β´. Further mutagenesis studies have shown that the C-terminal domain of Rio1 is essential and sufficient for this interaction [28]. Moreover, with the use of mutant yeast strains, the authors showed Rio1 as a target of CK2 in vivo, which was phosphorylated mainly by a CK2 heterotetramer containing both CK2α and CK2α’ subunits. In vitro kinase assays revealed a total of six clustered serine residues as the CK2 phosphorylation sites of yeast Rio1 (Figure 2) [28].

Although the physiologically important CK2-mediated phosphorylation of Rio1 affects the serine cluster, which is present exclusively in the yeast kinase (Figure 2) and has been proven to date in yeast cells only, it is the relevant starting point for further studies on regulation of the Rio1 via CK2-mediated phosphorylation.

## 4. Regulation of Rio1 via CK2-Mediated Phosphorylation in Yeast

Studies in yeast have revealed that CK2-mediated Rio1 phosphorylation can stimulate the activity of the atypical kinase in vitro. Six serines (S402, S403, S409, S416, S417, and S419) were identified as CK2 phosphorylation sites by means of site-directed mutagenesis. Consecutive mutation of the listed residues resulted in a decreased phosphorylation level, up to total abrogation of the signal in the case of mutation of all six serines. A Rio1 mutant mimicking the permanently phosphorylated enzyme, with the serines replaced with aspartic acids, showed approximately two fold higher phosphorylation activity toward histone H2B in comparison with the wild-type kinase and a Rio1 mutant lacking CK2-phosphorylated sites (S > A). In order to examine the possible biological importance of the in vitro studies, yeast cells harbouring a Rio1 mutant lacking serines modified by CK2 or a Rio1 mutant mimicking the state of permanent CK2-mediated phosphorylation were employed. The former yeast cells showed a significantly reduced growth rate, while the latter cells behaved indistinguishably from the wild-type yeast. The results show that phosphorylation of CK2-modified serines is essential for full activity of the kinase Rio1, and lack of the phosphorylation is disadvantageous for cell proliferation. Further, the authors revealed that the yeast cells harbouring the Rio1 mutant, which are not phosphorylated by CK2, had difficulties entering the S phase. Moreover, it has been shown that the S > A mutant shows low susceptibility to proteolytic degradation in contrast to the wild-type Rio1 and the S > D mutant [28].

In other studies, recombinant tobacco CK2-mediated phosphorylation of Rio1 from *Nicotiana tabacum* inhibited the interaction between Rio1 and the Movement Protein from *Tomato Mosaic Virus*. The authors suggest that phosphorylation occurs in the region of protein-protein interaction, and the process disrupts protein binding through changes in the protein conformation of Rio1 [25].

## 5. Collective Inhibition of CK2 and Rio1 by Benzimidazole Derivatives

Although the possible role of Rio1 in pathogenesis is not well established in comparison to many other kinases, it has been reported that the enzyme is upregulated in colon cancer, and there is a direct link between deregulation of ribosome biogenesis and tumor development [16]. In other studies, Read et al. showed that Rio1 and Rio2 were overexpressed in glioblastoma cells in an Akt-dependent manner and promoted tumorigenesis [29]. Taking this and the essential role of the Rio1 into consideration, the enzyme appears to be an attractive target in the fight against cancer. Therefore, selective and potent inhibitors of Rio1 are needed.

Currently there are not selective compounds targeting Rio1. The first small-molecule inhibitor of protein kinase Rio1 discovered is an antibiotic toyocamycin (TOYO). It is capable of inhibiting both ribosome biogenesis and kinase activity of Rio1 [10]. The antibiotic binds more tightly to Rio1 than ATP and exhibits mixed inhibition of the enzyme. Toyocamycin affects the kinase by stabilizing its less active oligomeric form. In other studies, a series of pyridine caffeic acid benzyl amides (CABA) were tested for their ability to inhibit the autophosphorylation activity of Rio1 [30]. Although three promising chemicals have been found, their activity starting from IC_50_ of 48 µM (AG490), at the ATP concentration of 1 µM which is optimal for Rio1, is rather low.

Derivatives of benzimidazoles are widely reported inhibitors of protein kinase CK2 from different sources, which show pro-apoptotic properties in tests on cancer cell lines and mouse xenografts [6,31,32,33,34,35]. While most benzimidazole chemicals show the highest potency against CK2, many of them can also affect other protein kinases, e.g., PIM, DYRK, or HIPK [31].

Our recent studies have revealed that Rio1 is inhibited in vitro by selected benzimidazoles with potency similar to that of CK2 (Table 1). The compounds with nanomolar activity against Rio1 appeared to be the most potent in vitro inhibitors of the kinase identified to date [9].

**Figure 3:**
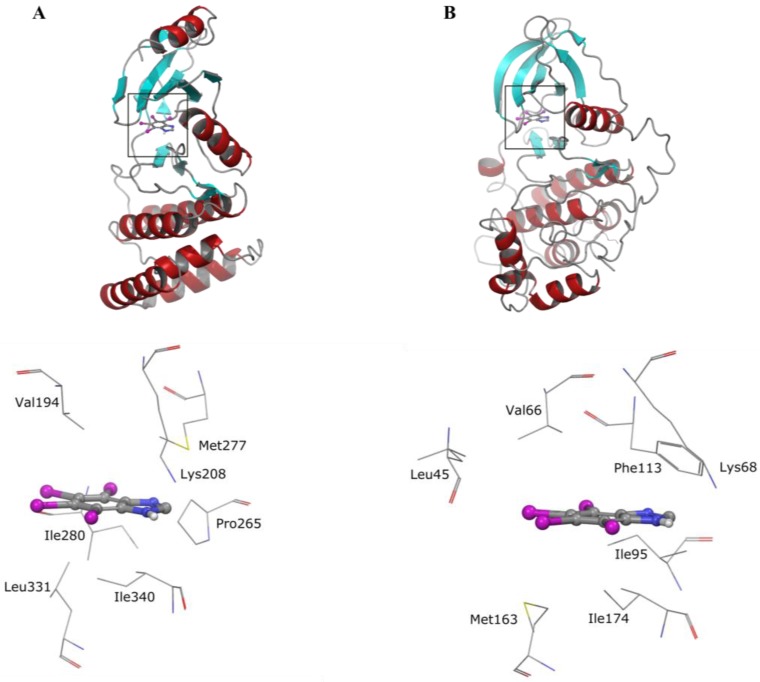
Docked binding modes obtained for TIBI in the ATP-binding pocket of (**A**) Rio1 and (**B**) CK2 [9].

Interestingly, Rio1 has not been tested before with other kinases in studies on the selectivity of benzimidazole inhibitors of CK2. Our data have shown that the most potent compound, i.e., TIBI, can act as a strict ATP-competitive inhibitor of the atypical kinase. Since the first inhibitor of Rio1 discovered, i.e., toyocamycin was reported to stabilize the less catalytically active oligomer and to enhance the thermostability of the kinase, we attempted to verify if these phenomena are general properties of the ATP-competitive inhibitors of Rio1. The results obtained have shown that TIBI does not influence the ternary structure of Rio1 but enhances the thermostability of the kinase. Molecular docking calculations have revealed that TIBI binds to the ATP-binding pockets of both kinases in a similar manner (Figure 3).

Although the nanomolar activity of some benzimidazoles against Rio1 could be a promising starting point in designing novel inhibitors of the kinase, the fact that these compounds target also many other kinases creates a serious barrier to reaching reasonable selectivity. On the other hand, taking into consideration the activity of toyocamycin against Rio1 and its selectivity with respect to CK2, the derivatization of the antibiotic appears to be a more attractive approach in designing potent and selective Rio1 inhibitors.

## 6. Conclusions

Summing up, there are some relationships between Rio1 and CK2. The enzymes can interact with one another. Rio1 undergoes CK2-mediated phosphorylation and this modification can regulate Rio1 activity. Although the articles reviewed here clearly show the importance of CK2 activity in Rio1 regulation, further studies should be carried out, mainly on human cells, in order to elucidate this interaction. However, the most intriguing aspect of their relationship is the shared susceptibility to benzimidazole inhibitors. This issue should be taken into consideration when new benzimidazole-based biologically active chemicals are designed. Considering the limited selectivity of benzimidazoles, the chemicals are not promising candidates for selective Rio1 inhibitors. However, benzimidazoles targeting a variety of kinase overexpressed in some pathological states, are widely used as anti-kinases agents. The novel anti-Rio1 activity of benzimidazoles discovered recently should be taken into consideration whenever new compounds are tested against a panel of protein kinases, and in such a case the atypical protein kinase Rio1 cannot be disregarded.

## Figures and Tables

**Table 1:** Inhibitors of protein kinase Rio1 [9,10,30].

TIBI	K92	DMAT	TBI	TBB	TCI	TOYO	AG490
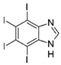	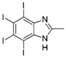	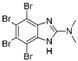	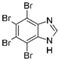	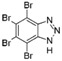	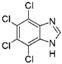	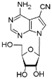	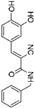
Rio1, IC_50_ [µM]							
0.09	0.19	0.19	0.33	1.74	1.9	3.66	47.88
CK2, IC_50_ [µM]							
0.083	0.066	0.19	0.44	0.19	9.7	54.78	n.d. *

*n.d.—not determined.
